# Safety Assessment of Lemon Myrtle (*Backhousia citriodora*) Extract: 28-Day Oral Toxicity Study in Rats and In Vitro and In Vivo Genotoxicity Studies

**DOI:** 10.3390/toxics14030213

**Published:** 2026-02-28

**Authors:** Takashi Yamaguchi, Shinichi Honda, Toshihide Fujii, Ayumi Yamamoto, Keiichi Itoh, Maya Ueda, Shoji Masumori, Hiroshi Kubo

**Affiliations:** 1 Pharmacology & Toxicology Research Term, Bio-Pharma Research Laboratories, Kaneka Corporation, Takasago 676-8688, Japan; toshihide.fujii@kaneka.co.jp (T.F.); hiroshi.kubo@kaneka.co.jp (H.K.); 2 Supplement & Probiotics Research Laboratories, Kaneka Corporation, Takasago 676-8688, Japan; shinichi.honda@kaneka.co.jp (S.H.); ayumi.yamamoto@kaneka.co.jp (A.Y.); 3 Iwata Research Institute, Trans Genic Inc., Iwata 437-1213, Japan; keiichi.itoh@transgenic.co.jp (K.I.); maya.ueda@transgenic.co.jp (M.U.); shoji.masumori@transgenic.co.jp (S.M.)

**Keywords:** food, genotoxicity, lemon myrtle, toxicity, safety

## Abstract

The essential oil or extract of Lemon myrtle (*Backhousia citriodora* F. Muell.), belonging to the family Myrtaceae and the genus *Backhousia*, exhibits anti-inflammatory and antioxidant properties. However, limited information exists on the safety of water extracts from its leaves. The present study aimed to assess the safety of lemon myrtle water extract as a functional food by performing genotoxicity studies and repeated-dose oral toxicity. Although the bacterial reverse mutation test (Ames test) yielded positive results, in vivo mammalian erythrocyte micronucleus and alkaline comet assays yielded negative results. In a 28-day oral toxicity study, the extract was orally administered to male and female Crl:CD rats at doses of 0, 250, 500, and 1000 mg/kg bw/day. Notably, the extract induced no adverse effects, and the no-observed-adverse-effect level was 1000 mg/kg bw/day in male and female rats. Despite its genotoxicity in vitro, the extract did not exhibit genotoxicity in vivo. Moreover, no signs of toxicity were observed in the general toxicity study. Overall, these results suggest that lemon myrtle water extract does not pose a substantive genotoxic risk at practical oral exposure levels.

## 1. Introduction

With the recent increasing demand for functional foods, it becomes important to evaluate their clinical safety. Lemon myrtle (*Backhousia citriodora* F. Muell.) belongs to the family Myrtaceae and is native to Queensland on the east coast of Australia [1]. The essential oil derived from lemon myrtle leaves is a volatile extract containing predominantly citral (90–97%) [2]. Commonly incorporated as a flavoring and aromatic ingredient in foods and cosmetics, citral is also used in aromatherapy massage oils and other household products such as detergents, soaps, air fresheners, and insect repellents [3]. In addition, the bioactivity of citral is well-established. Lemon myrtle essential oil or extract exhibits anti-inflammatory and antioxidant properties, as well as antimicrobial effects against foodborne and postharvest pathogens and skin infections [3,4,5,6,7]. The genotoxic potential of citral is considered negative because seven bacterial reverse mutation tests (Ames test), two chromosomal aberration tests, and two in vivo micronucleus tests in rodents have yielded negative results; however, one sister chromatid exchange test yielded positive results [8]. In addition, the no-observed-adverse-effect level (NOAEL) for developmental toxicity is 200 mg/kg bw/day of oral citral, which causes skin irritation but not eye irritation in rabbits and induces skin sensitization in guinea pigs [8,9]. Notably, citral is included in the Generally Recognized as Safe list by the U.S. Food and Drug Administration [3].

Lemon myrtle leaf water extract (LME) exhibits antimicrobial activity against *Staphylococcus aureus* [10] and inhibits both glucosyltransferase activity and lactate production in *Streptococcus mutans* [11,12]. LME contains casuarinin, a component reported to promote the activation of skeletal muscle satellite cells in vitro and in vivo, which suggests that LME could be developed as a novel nutritional intervention for sarcopenia [13]. In clinical study, intake of LME was found to enhance the muscle hypertrophy effect of resistance training [14]. As the composition and content of plant extracts vary depending on the extraction method, it is necessary to evaluate the extracted components and determine the safety of each extract as a functional food. To date, no studies have evaluated the genotoxicity and repeated oral toxicity of a water extract of lemon myrtle leaves prepared using the present extraction method.

As food products are expected to be consumed by humans over the long term, evaluating their genotoxicity is important for assessing potential carcinogenic risks. The Ames and in vivo mammalian erythrocyte micronucleus tests are widely used for detecting genetic mutations, including base substitutions and frameshifts, and structural or numerical chromosome damage induced by the test components, respectively. The in vivo alkaline comet assay is used to detect initial DNA damage caused by test compounds. It is highly sensitive and specific. Moreover, by accounting for various in vivo metabolic and pharmacokinetic factors, it offers valuable insight into mutagenicity and genotoxicity [15]. Another in vivo genotoxic test system is the in vivo transgenic rodent mutation (TGR) assay, which can detect genetic mutations. It is superior as a follow-up test to an in vitro positive result. However, the working group on in vivo genotoxicity testing strategies for the 7th International Workshop on Genotoxicity Testing reported that the in vivo mammalian alkaline comet assay, conducted in the liver or gastrointestinal tract, is equally suitable as the in vivo TGR assay [16]. In the present study, we aimed to comprehensively assess the safety of a lemon myrtle water extract, which comprises LME and dextrin, by conducting genotoxicity tests following International Council for Harmonisation of Technical Requirements for Pharmaceuticals for Human Use S2 (R1) (Guidance on Genotoxicity Testing and Data Interpretation for Pharmaceuticals Intended for Human Use), including the Ames test, the in vivo micronucleus test, and the in vivo mammalian alkaline comet assay. Additionally, we performed a 28-day repeated oral toxicity test in rats as a general toxicity test.

## 2. Materials and Methods

### 2.1. Preparation of Test Substance

Cut and dried lemon myrtle leaves (Australian Native Lemon Myrtle Farms, Airlie Beach, Australia; Lot Nos. 1116 and 1216) were used as the starting material. The leaves were extracted with water (eightfold volume) at 80 °C for 1 h and the suspension was filtered using a filter press with diatomite as a filter aid. The clear supernatant (filtrate) was collected and concentrated under reduced pressure. The concentrated extract (LME; the active ingredient), along with dextrin Max 1000 (Matsutani Chemical Industry, Itami, Japan) as an excipient, was heat-sterilized and spray-dried to obtain a dry powder (Lot No. LME-N-T02, refrigeration storage). This powder served as the test substance used in all studies. It comprised 83 *w*/*w*% LME and 17 *w*/*w*% dextrin Max 1000, and the reported amounts and dosages refer specifically to the LME content. The quantitative composition of the major compounds of LME has already been determined by high-performance liquid chromatography using extracts prepared by the same manufacturing process; the analytical conditions (including column specifications and elution parameters) and results have been reported. The results are as follows: based on dry weight content, the active constituents were casuarinin (1.61 *w*/*w*%), quercitrin (3.88 *w*/*w*%), hyperin (1.91 *w*/*w*%), myricitrin (1.26 *w*/*w*%), and gallic acid (0.25 *w*/*w*%) [13]. The stability of major constituents was confirmed in a separate stability study conducted over the same period as the experimental study, with no significant changes observed in the main constituents. The nutritional composition of the test substance was analyzed in accordance with the methods specified in the Food Labeling Standards [17], and the results were as follows: water (3.5 *w*/*w*%), protein (1.2 *w*/*w*%), fat (0.4 *w*/*w*%), total carbohydrates (86.1 *w*/*w*%, including 1.4 *w*/*w*% dietary fiber), and ash (8.8 *w*/*w*%).

### 2.2. Animals

Seven-week-old male Crl:CD (Sprague Dawley [SD]) rats (for the alkaline comet assay) and five-week-old male and female Crl:CD (SD) rats (for the 28-day oral toxicity study) were purchased from Jackson Laboratory Japan Inc. (Atsugi, Japan). Eight-week-old male B6D2F1 mice (for the mammalian erythrocyte micronucleus test) were purchased from Japan SLC Inc. (Hamamatsu, Japan). Upon receipt, the animals were quarantined and acclimated to the experimental environment for at least 7 days before initiating each experiment. All animals were maintained in a barrier-controlled animal facility and housed individually or in pairs in wire-mesh cages mounted on an automatic water-flushing rack system (Tokyo, Japan). Environmental conditions were controlled, with the room temperature maintained at 20–26 °C and relative humidity at 35–70% under a 12 h light/dark cycle and a ventilation rate of at least 12 air changes per hour. Animals had free access to a radiation-sterilized commercial diet (CRF-1; Oriental Yeast, Tokyo, Japan) and municipal tap water from Iwata via an automated watering system. Analytical assessments confirmed that contaminant levels in feed and water were within acceptable limits.

All the experimental protocols were reviewed and approved by the Institutional Animal Care and Use Committee of the Iwata Research Institute, Trans Genic Inc. (Iwata, Japan). The approval numbers are 16-0302A for the in vivo mammalian erythrocyte micronucleus test, 17-0106A for the in vivo alkaline comet assay, and 16-0299A for the 28-day oral toxicity study. The experiments were conducted in compliance with the Act on the Welfare and Management of Animals, Standards Relating to the Care and Management of Laboratory Animals, and the Relief of Pain and Guidance for Animal Testing of the Iwata Research Institute, Trans Genic Inc.

### 2.3. Genotoxicity Studies

All genotoxicity studies were conducted in compliance with the Organization for Economic Co-operation and Development (OECD) Principles of Good Laboratory Practice [18] and the Ministerial Ordinance on Good Laboratory Practice for Nonclinical Safety Studies of Drugs [19].

#### 2.3.1. Bacterial Reverse Mutation Test (Ames Test)

The Ames test was carried out in accordance with OECD Test Guideline 471 [20] using the pre-incubation method (37 °C, 20 min) and the Guidelines for Designation of Food Additives and Revision of Standards for Use of Food Additives [21].

The test used *Salmonella typhimurium* strains TA98, TA100, TA1535, and TA1537, originally sourced from Dr. Bruce N. Ames (University of California, Berkeley, CA, USA), and *Escherichia coli* WP2*uvrA*, supplied by the National Institute of Hygienic Sciences (Japan; now the National Institute of Health Sciences).

Distilled water was used as the negative control. For the pre-incubation method conducted without metabolic activation, the following positive controls were used: 2-(2-furyl)-3-(5-nitro-2-furyl)acrylamide (AF-2) at 0.01 µg/plate for TA100 and WP2*uvrA*, AF-2 at 0.1 µg/plate for TA98, sodium azide (NaN_3_) at 0.5 µg/plate for TA1535, and 9-aminoacridine hydrochloride (9-AA) at 80 µg/plate for TA1537. With metabolic activation, 2-aminoanthracene (2-AA) was used as the positive control at concentrations of 0.5 µg/plate for TA98, 1.0 µg/plate for TA100, 2.0 µg/plate for TA1535 and TA1537, and 10 µg/plate for WP2*uvrA*. AF-2, NaN_3_, and 2-AA were purchased from Wako Pure Chemical Industries (Osaka, Japan) and 9-AA was obtained from Sigma-Aldrich (St. Louis, MO, USA).

Metabolic activation was provided by an S9 microsomal fraction prepared from the livers of Sprague Dawley rats induced with phenobarbital and 5,6-benzoflavone (Ames Test-S-9/Cofactor A Set; Oriental Yeast, Tokyo, Japan). The resulting S9 mixture (10 *v*/*v*% as S9) was sterile.

A preliminary study was conducted in duplicates to investigate bacterial growth inhibition and mutagenicity of LME at final concentrations of 8.19, 20.5, 51.2, 128, 320, 800, 2000, and 5000 µg/plate, in the absence and presence of metabolic activation. The number of revertant colonies in the LME-treated group, both in the absence and presence of S9, in the TA98 strain at 5000 µg/plate exceeded twice that observed in the negative control group, and a concentration-dependent increase was observed. In the absence of S9, growth inhibition was observed at concentrations of 2000 µg/plate or higher for the TA1537 strain and at 5000 µg/plate for the TA100, TA1535, WP2*uvrA*, and TA98 strains. In contrast, no growth inhibition was observed in any strain in the presence of S9. Moreover, LME precipitation was observed 5000 µg/plate in the absence of S9 and at concentrations of 2000 µg/plate or higher in the presence of S9. Based on the preliminary results, except in the absence of metabolic activation of strain TA1537, the main study was conducted in duplicate with final LME concentrations of 156, 313, 625, 1250, 2500, and 5000 µg/plate, in the absence and presence of metabolic activation. In the absence of metabolic activation of strains TA1537, the main study was conducted in duplicate with final LME concentrations of 78.1, 156, 313, 625, 1250, and 2500 µg/plate. Positive controls were included in all the tests.

Revertant colonies were enumerated using an automated colony analyzer (CA-11; System Sciences, Tokyo, Japan); the results for the test article were evaluated relative to the reference control. Each plate was inspected microscopically for LME precipitation at the beginning and end of the exposure period. A response was judged positive when the mean revertant count on the treated plates showed a reproducible, dose-related increase of at least 2-fold compared with the corresponding control values.

#### 2.3.2. In Vivo Mammalian Erythrocyte Micronucleus Test

The in vivo mammalian erythrocyte micronucleus test was carried out in accordance with OECD Test Guideline 474 [22] and the Designation of Food Additives and Revision of Standards for Use of Food Additives [21].

Thirty male B6D2F1 mice were divided into five groups of six mice each. LME was orally administered consecutively for 2 days (24-h intervals) at 0 (negative control, distilled water), 500, 1000, and 2000 mg/kg bw/day. The positive control group received a single oral dose of 25 mg/kg bw/day cyclophosphamide (CP, Shionogi & Co., Ltd., Osaka, Japan) dissolved in saline (Japanese Pharmacopoeia, Otsuka Pharmaceutical Factory, Inc., Tokushima, Japan). The animals were administered a dose of 10 mL/kg.

General conditions of the animals in the LME-treated and negative control groups were monitored 1, 24, 25, and 48 h after the initial administration. Body weights were recorded prior to euthanasia. In the positive control group, general conditions were assessed 1 h after dosing and again immediately before bone marrow collection (24 h after administration). Animals in the LME-treated and negative control groups were euthanized by CO_2_ inhalation 24 h after the second administration, whereas positive control animals were euthanized 24 h after cyclophosphamide administration. The femurs were excised and bone marrow cells were flushed with heat-inactivated calf serum (56 °C for 30 min; Life Technologies Corporation, Grand Island, NY, USA), centrifuged to remove excess serum, and resuspended in Dulbecco’s phosphate-buffered saline (Sigma-Aldrich). Following methanol fixation, the cells were stained with 3% Giemsa. Three bone marrow smears per animal were prepared from all six animals in each group and randomly coded. Microscopic scoring was then carried out for five animals per group in ascending order of animal identification numbers. For each animal, 2000 polychromatic erythrocytes (PCEs) were examined and micronucleated PCEs (MNPCEs) were enumerated. To assess potential bone marrow toxicity, the proportion of PCEs was determined by counting PCEs among 500 total erythrocytes.

#### 2.3.3. In Vivo Mammalian Alkaline Comet Assay

The in vivo mammalian alkaline comet assay was carried out in accordance with OECD Test Guideline 489 [23].

Thirty male Crl:CD (SD) rats were divided into five groups of six rats each. LME was orally administered consecutively for 2 days (21-h intervals) at 0 (negative control, distilled water), 500, 1000, and 2000 mg/kg bw/day. Ethyl methanesulfonate (Sigma-Aldrich) was orally administered as a positive control at a dose of 200 mg/kg bw/day and a dosing volume of 10 mL/kg.

General conditions were monitored 1 h after the first administration, at 21 h (prior to the second administration), at 22 h (1 h after the second administration), and at 24 h (immediately before necropsy). Body weights were recorded immediately before necropsy. Because no target organs were identified in the 28-day repeated oral toxicity study (Section 2.4), the glandular stomach (the initial site of exposure) and liver (a major organ for metabolism) were selected for assessment.

Animals were euthanized by CO_2_ inhalation 3 h after the second administration, and the glandular stomach and liver were collected. The organs were subsequently washed with a homogenization buffer composed of Hanks’ balanced salt solution (Life Technologies) supplemented with 25 mmol/L ethylenediaminetetraacetic acid disodium salt (EDTA·2Na; Dojindo Laboratories, Kumamoto, Japan) and 10% (*v*/*v*) dimethyl sulfoxide (Wako Pure Chemical Industries), adjusted to pH 7.5. After rinsing, tissues were examined macroscopically for gross abnormalities. The glandular stomach and selected portions of the left lateral liver lobe were excised and processed to obtain single-cell suspensions; additional samples were collected for histopathological evaluation.

For each tissue, three comet assay slides were generated (two designated for scoring and one retained as a backup). To cast the gels, 10 µL of the single-cell suspension was combined with 90 µL of 0.5% low-melting-point agarose and applied to the slides. The slides were equilibrated in alkaline electrophoresis buffer (pH > 13) composed of 300 mmol/L NaOH (Kanto Chemical, Tokyo, Japan) and 1 mmol/L EDTA·2Na (Dojindo Laboratories) and then placed in a submarine-type electrophoresis unit (BE-540). Ice-cold buffer was carefully added to fully immerse the gels and the slides were left for 20 min to allow DNA unwinding. Electrophoresis was conducted for 20 min at 25 V (0.7 V/cm), starting at an initial current of 300 mA. After electrophoresis, the slides were stained with SYBR Gold (SYBR Gold nucleic acid gel stain; Life Technologies, Grand Island, NY, USA) for fluorescence-based visualization.

For each group, slides from five animals were assessed under a fluorescence microscope (Olympus Life Science Solutions, Tokyo, Japan) in ascending order of animal ID. For each organ, 150 cells per animal were scored (75 cells per slide; 750 cells per group). DNA migration images were acquired using a CCD camera coupled to the microscope, imported into a computer, and quantified using comet analysis software (version 4.3.2; Comet Assay IV System; Perceptive Instruments, Instem Group of Companies, Stone, UK). Hedgehogs were counted separately in an additional 150 cells per animal (75 cells per slide) using fluorescence microscopy.

The percentage of tail DNA compared to total DNA (% tail DNA: tail % intensity) was used as an indicator of DNA damage. The median % tail DNA of each slide was used as the slide value, and the animal value represented the mean of the slide values for each animal.

As the comet assay did not reveal DNA damage in any of the organs, they were not histopathologically examined.

#### 2.3.4. Statistical Analyses

In the Ames test, no statistical analyses were performed. In the in vivo mammalian erythrocyte micronucleus assay, MNPCE frequencies in each treatment group, including the positive control, were compared with the negative control using the conditional binomial approach of Kastenbaum and Bowman (upper *p* = 0.025). The proportion of PCEs among total erythrocytes was computed and analyzed using Dunnett’s multiple comparison test (two-sided, *p* = 0.05) to compare the negative control and LME-treated groups. The Aspin–Welch *t*-test (two-sided, *p* = 0.05) was used to compare the negative and positive control groups. Results were considered positive when the MNPCE frequency differed significantly from that of the negative control.

For the in vivo mammalian alkaline comet assay, animal-level mean % tail DNA values were log-transformed and compared between LME-treated groups and the negative control using Dunnett’s multiple comparison test (two-sided, *p* = 0.05). The positive control group was compared with the negative control using the Aspin–Welch *t*-test with a one-sided significance level of 0.025.

### 2.4. 28-Day Oral Toxicity Study

This study was conducted in accordance with OECD Test Guideline 407 [24], the OECD Principles of Good Laboratory Practice [18], the Ministerial Ordinance on Good Laboratory Practice for Nonclinical Safety Studies of Drugs [19], and the Guidelines for Designation of Food Additives and Revision of Standards for Use of Food Additives [21].

#### 2.4.1. Study Design

Twenty male and twenty female Crl:CD (SD) rats were divided into four groups of five rats from each sex. Starting day 1 of treatment, LME was orally administered for 28 consecutive days at 0 (vehicle control, distilled water, Otsuka Pharmaceutical Factory, Inc., Tokushima, Japan), 250, 500, and 1000 mg/kg bw/day at a volume of 10 mL/kg. The dose was calculated based on the latest body weight measurements. At the end of the treatment period, after overnight fasting with ad libitum access to water, the rats were necropsied by exsanguination under isoflurane anesthesia and then examined macroscopically.

#### 2.4.2. General Conditions, Body Weight, and Food Consumption

All animals were observed for general condition and mortality twice daily (before and after dosing). On the day of necropsy, general conditions were recorded once. Body weights were measured weekly throughout the dosing period and again on the day of necropsy (day 29) following an overnight fast.

Food consumption was recorded weekly on the same days as body weight measurements and mean daily food consumption was calculated.

#### 2.4.3. Ophthalmologic Examinations and Urinalysis

Ophthalmological examinations were conducted among all animals in the control and the 1000 mg/kg bw/day groups during the acclimation (day −2) and treatment (day 27) periods. These observations included observing the appearance and light reflex and examining the anterior segment of the eyeball, optic media, and fundus oculi.

All animals were placed in urine collection cages under feeding and watering conditions during the acclimation period (from days −4 to −3) and treatment period (from days 26 to 27), and fresh (4 h) and accumulated (24 h) urine were collected. Urinalysis was conducted using Ames test strips (N-Multistix SG-L; Siemens Healthcare Diagnostics, Eschborn, Germany) and an automatic strip reader (CLINITEK Advantus; Siemens Healthcare Diagnostics). The following parameters were assessed: pH, occult blood, ketone bodies, glucose, protein, bilirubin, and urobilinogen. Urine volume and color were also examined. Urinalysis parameters, examined following the centrifugation (400× *g* for 5 min) of urine samples, included sediment, osmotic pressure (AUTO&STATTM OM-6030; Arkray Factory, Kyoto, Japan), and the concentrations of sodium, potassium, and chloride ions (electrolyte analyzer EA07; A&T, Kanagawa, Japan). Urine sediments were examined microscopically.

#### 2.4.4. Hematology and Serum Biochemistry

Prior to necropsy, whole blood samples were obtained from the abdominal aorta under isoflurane anesthesia for hematological, coagulation, and serum biochemical analyses. For hematological assessments, blood was collected into tubes containing dipotassium ethylenediaminetetraacetic acid. Hematological parameters were measured using an automated analyzer (ADVIA120; Siemens Healthcare Diagnostics) or derived from the measured values, including hematocrit (HCT), hemoglobin concentration, red blood cell count (RBC), mean corpuscular volume (MCV), mean corpuscular hemoglobin (MCH), mean corpuscular hemoglobin concentration (MCHC), reticulocyte concentration (RETIC), platelet count, white blood cell count (WBC), and leukocyte differential counts (neutrophils, lymphocytes, monocytes, eosinophils, basophils, and large unstained cells).

For coagulation testing, blood was collected into tubes containing sodium citrate (3.13% solution) and centrifuged at 1700× *g* for 13 min to obtain plasma. Coagulation parameters, including prothrombin time (PT), activated partial thromboplastin time (APTT), and fibrinogen concentration, were determined using a coagulation analyzer (STA Compact; Roche Diagnostics, Basel, Switzerland).

For serum biochemistry, blood was collected into tubes containing a gel separator and clot activator and then centrifuged at 1700× *g* for 7 min to obtain serum. Serum biochemical parameters were quantified using an automated analyzer (Hitachi 7170; Hitachi High-Tech, Tokyo, Japan): glucose, triglyceride, total cholesterol, blood urea nitrogen, creatinine, total bilirubin, aspartate aminotransferase, alanine aminotransferase, alkaline phosphatase, γ-glutamyl transpeptidase activity, calcium, inorganic phosphorus, and total protein. The following parameters were determined using an electrophoresis analyzer (Epalyzer 2 plus; Helena Laboratories, Beaumont, TX, USA): albumin, α1-, α2-, β-, and γ-globulin ratios. The concentration of each protein fraction and albumin-to-globulin ratios were calculated by measuring the ratio of each protein fraction. Sodium, potassium, and chloride concentrations were determined using an electrolyte analyzer (EA07; A&T).

#### 2.4.5. Organ Weights, Macroscopic Examination, and Histopathology

All animals were euthanized by exsanguination after blood collection under isoflurane anesthesia. They were then subjected to organ weight, macroscopic, and histopathological examinations, as specified in the OECD test guidelines.

Relative organ weights (organ-to-body weight ratios) were calculated using the body weight recorded on the day of necropsy. For histopathological examination, organs and tissues were fixed in 10% neutral-buffered formalin. Testes and eyes (including the optic nerves and Harderian glands) were pre-fixed in formalin–acetic acid solution and Davidson’s solution, respectively, prior to routine fixation. Fixed tissues were then paraffin-embedded, sectioned, and stained with hematoxylin and eosin. Histopathological examinations were performed on the following specimens in the control and 1000 mg/kg bw/day groups: lung (including bronchi), heart, intrathoracic aorta, kidneys, liver, thymus, spleen, pancreas, lymph nodes (cervical and mesenteric), adrenals, salivary glands (sublingual and submandibular glands), pituitary, brain (excluding the olfactory bulb), spinal cord (cervical, thoracic, and lumbar regions), tongue, forestomach, glandular stomach, duodenum, jejunum, ileum (including Peyer’s patches), cecum, colon, rectum, testes, epididymides, seminal vesicles (including coagulating glands), prostate (including urethra), ovaries, oviduct, uterus, vagina, urinary bladder, eye (including the optic nerve), Harderian gland, skeletal muscle (thigh), sciatic nerve, skin, mammary gland, trachea, esophagus, thyroid gland, parathyroid gland, sternum, femur, bone marrow (sternum and femur), Zymbal’s gland, and nasal cavity (nasal turbinates). In the male 250 and 500 mg/kg bw/day groups, histopathological examination was performed only on the kidneys. All findings were graded according to severity.

#### 2.4.6. Statistical Analyses

Data are presented as mean ± standard deviation. Statistical analyses were performed using LATOX-F software (version 5; Fujitsu Limited, Tokyo, Japan). Homogeneity of variance was assessed using Bartlett’s test for body weight, body weight gain, food consumption, hematological parameters, coagulation parameters, serum biochemistry, serum protein electrophoresis, urinalysis (urine volume, osmotic pressure, and electrolytes), and absolute and relative organ weights. When the variance was homogeneous, Dunnett’s multiple comparison test was applied to compare each treatment group with the control group. For data with heterogeneous variance, Steel’s test was applied. Bartlett’s test was evaluated at a 5% significance level, whereas other statistical tests were performed using two-sided analyses at significance levels of 5% and 1%.

## 3. Results

### 3.1. Genotoxicity Studies

#### 3.1.1. Bacterial Reverse Mutation Test (Ames Test)

The results of the Ames tests are presented in Table 1. Consistent with the results of the preliminary study, in the main study, the number of revertant colonies in the LME-treated group, both in the absence and presence of S9 in the TA98 strain at 5000 µg/plate, was more than twice that in the negative control group. Moreover, a concentration-dependent increase was observed. In the absence of S9, growth inhibition of the test strains was observed at a concentration of 1250 µg/plate or higher for the TA1537 strain, at 2500 µg/plate or higher for TA1535 and TA98 strains, and at 5000 µg/plate for the TA100 and WP2*uvrA* strains. In contrast, no growth inhibition was observed in any strain in the presence of S9. Moreover, LME precipitation was observed at a concentration of 5000 µg/plate in the absence of S9 and at 2500 µg/plate or higher in the presence of S9.

Compared to the negative control group, all strains in the positive control group exhibited a two-fold or greater increase in the number of revertant colonies, which were all within the criteria of the laboratory background data, thus validating the method.

**Table 1:** Results of the main bacterial reverse mutation test conducted with LME.

Test Substance	Concentration	Mean Revertant Colonies Per Plate (Mutagenic Index Is Indicated in Parentheses)									
	(µg/Plate)	TA100		TA1535		WP2*uvrA*		TA98		TA1537	
		-S9	+S9	-S9	+S9	-S9	+S9	-S9	+S9	-S9	+S9
Distilled Water ^a^	0	129	129	13	14	27	24	24	31	7	15
		(1.00)	(1.00)	(1.00)	(1.00)	(1.00)	(1.00)	(1.00)	(1.00)	(1.00)	(1.00)
LME	78.1	-	-	-	-	-	-	-	-	8	-
		-	-	-	-	-	-	-	-	(1.14)	-
	156	129	136	16	18	27	21	26	30	4	21
		(1.00)	(1.05)	(1.23)	(1.29)	(1.00)	(0.88)	(1.08)	(0.97)	(0.57)	(1.40)
	313	104	124	18	14	21	29	29	28	8	21
		(0.81)	(0.96)	(1.38)	(1.00)	(0.78)	(1.21)	(1.21)	(0.90)	(1.14)	(1.40)
	625	102	118	13	14	25	24	24	36	7	19
		(0.79)	(0.91)	(1.00)	(1.00)	(0.93)	(1.00)	(1.00)	(1.16)	(1.00)	(1.27)
	1250	108	135	14	14	19	30	32	43	6 *	22
		(0.84)	(1.05)	(1.08)	(1.00)	(0.70)	(1.25)	(1.33)	(1.39)	(0.86)	(1.47)
	2500 ^e^	105	133	14 *	12	21	25	37 *	49	6 *	17
		(0.81)	(1.03)	(1.08)	(0.86)	(0.78)	(1.04)	(1.54)	(1.58)	(0.86)	(1.13)
	5000 ^d,e^	118 *	126	15 *	16	22 *	14	48 *	67	-	19
		(0.91)	(0.98)	(1.15)	(1.14)	(0.81)	(0.58)	(2.00)	(2.16)	-	(1.27)
Positive Control ^b,c^		510	950	653	340	100	1028	595	356	166	185
		(3.95)	(7.36)	(50.23)	(24.29)	(3.70)	(42.83)	(24.79)	(11.48)	(23.71)	(12.33)

2-AA, 2-aminoanthracene; 9-AA, 9-aminoacridine hydrochloride; AF-2, 2-(2-furyl)-3-(5-nitro-2-furyl) acrylamide; LME, Lemon myrtle leaf water extract; NaN_3_, Sodium azide. -S9, without metabolic activation; +S9, with metabolic activation. ^a^ Negative control: 100 µL/plate. ^b^ Positive controls -S9: TA100 and WP2*uvrA*; 0.01 µg/plate AF-2, TA1535; 0.5 µg/plate NaN_3_, TA98; 0.1 µg/plate AF-2, TA1537; 80 µg/plate 9-AA. ^c^ Positive controls +S9: TA100; 1.0 µg/plate 2-AA, TA1535 and TA1537; 2.0 µg/plate 2-AA, WP2*uvrA*; 10 µg/plate 2-AA, TA98; 0.5 µg/plate 2-AA. ^d^ Visible precipitation was observed at the end of the exposure period (-S9). ^e^ Visible precipitation was observed at the end of the exposure period (+S9). * Growth inhibition was observed. -, Not applicable.

#### 3.1.2. In Vivo Mammalian Erythrocyte Micronucleus Test

The results of the mammalian erythrocyte micronucleus assay are presented in Table 2. No clinical signs were observed in mice, and body weight gain was normal in all the groups.

There were no significant differences in the frequency of MNPCE between the LME-treated and negative control groups, whereas it was significantly higher in the positive control group than in the negative control group (*p* < 0.025). Compared to the negative control group, the LME-treated and positive control groups exhibited no significant differences in the PCE ratio.

**Table 2:** Results of the in vivo mammalian erythrocyte micronucleus test in male mice treated with LME.

Test Substance	Dose (mg/kg bw/Day)	No. of Mice	Frequency of MNPCE (%)	Range of MNPCE/2000 PCE (Min–Max)	Ratio of PCE (%)
Distilled Water ^a^	0	5	0.25 ± 0.07	4–7	52.2 ± 2.9
LME	500	5	0.22 ± 0.13	2–8	52.6 ± 3.6
	1000	5	0.25 ± 0.13	2–8	49.9 ± 4.9
	2000	5	0.25 ± 0.11	2–8	53.2 ± 5.8
Cyclophosphamide ^b^	25	5	1.55 ± 0.24 *	26–39	52.2 ± 2.4

Frequency of MNPCE (%) and ratio of PCE (%); data are represented as mean ± standard deviation. LME, Lemon myrtle leaf water extract; MNPCE, micronucleated polychromatic erythrocyte; PCE, polychromatic erythrocyte. ^a^ Negative control: 10 mL/kg, ^b^ Positive control. * Significant difference compared to the negative control (Kastenbaum and Bowman’s test, *p* < 0.025).

#### 3.1.3. In Vivo Mammalian Alkaline Comet Assay

The results of the in vivo mammalian alkaline comet assay are depicted in Table 3, and microscopic images of the liver are illustrated in Figure S1. In the LME-treated groups, no abnormalities were observed in the general condition or gross findings of the glandular stomach or liver, nor were there any changes in body weight.

There were no significant differences in the % tail DNA and the frequency of hedgehogs in the glandular stomach or liver cells between the LME-treated and negative control groups. In contrast, the % tail DNA was significantly increased in both the glandular stomach and liver cells in the positive control group compared to those in the negative control group (*p* < 0.025), and the % tail DNA in the negative control group was within the acceptable range of the historical data of the laboratory, thereby validating the study.

**Table 3:** Results of in vivo mammalian alkaline comet assay test in male rats treated with LME.

Test Substance	Dose (mg/kg bw/Day)	No. of Rats	Glandular Stomach		Liver	
			% Tail DNA ^a^	Frequency of Hedgehogs (%)	% Tail DNA	Frequency of Hedgehogs (%)
Distilled Water ^b^	0	5	3.93 ± 0.60	1.7 ± 0.6	0.79 ± 0.05	0.0 ± 0.0
LME	500	5	3.95 ± 0.34	0.5 ± 0.6	0.80 ± 0.10	0.5 ± 0.6
	1000	5	3.67 ± 0.71	0.5 ± 0.7	0.71 ± 0.11	0.3 ± 0.4
	2000	5	3.32 ± 0.31	0.8 ± 0.9	0.81 ± 0.11	0.3 ± 0.4
Ethyl methanesulfonate ^c^	200	5	30.41 ± 2.99 *	0.5 ± 0.6	24.18 ± 2.17 *	0.3 ± 0.4

Data are represented as mean ± standard deviation. LME, Lemon myrtle leaf water extract. ^a^ The mean % tail DNA in each group was calculated from that in each animal based on the median % tail DNA in each slide. ^b^ Negative control: 10 mL/kg, ^c^ Positive control. * Significant difference compared to the negative control (Aspin–Welch’s *t*-test, *p* < 0.025).

### 3.2. 28-Day Oral Toxicity Study

#### 3.2.1. General Conditions, Body Weight, and Food Consumption

No mortality or treatment-related abnormalities in general condition were observed in male or female rats administered LME. Body weight changes are shown in Figures S2 and S3 and food consumption changes are presented in Figure S4.

There were no significant differences in body weights between the LME-treated and control groups. Compared to the body weight gain in the control group, that in LME-treated male rats was not significantly different, whereas it was significantly decreased in the 1000 mg/kg bw/day LME-treated female rats (*p* < 0.05). However, this change in females was considered to be incidental and unrelated to LME administration, as there were no inter-day variations in body weights and no decrease in food intake.

On day 28, food consumption in the male 500 mg/kg bw/day group was significantly higher, whereas it was significantly lower in the female 250 mg/kg bw/day group than in the control group (*p* < 0.05). As these changes were negligible and dose-independent, they were presumed not to be caused by LME administration.

#### 3.2.2. Ophthalmologic Examination and Urinalysis

The ophthalmology and urinalysis data are presented in Tables S1 and S2, respectively. Ophthalmological examination on day 27 revealed corneal opacity in 3 and 1 animal in the male and female 1000 mg/kg bw/day groups, respectively. However, this finding, which was also observed in the control group, was considered to have low toxicological significance and was presumably unrelated to LME. On urinalysis from days 26 to 27, no significant test findings nor statistically significant differences were observed between the control group and any of the LME-treated male or female rats.

#### 3.2.3. Hematology and Serum Biochemistry

Hematological and serum biochemistry data are presented in Table 4 and Table 5, respectively. The PT was significantly shortened in the male 500 and 1000 mg/kg bw/day groups compared to that in the control group (*p* < 0.01). However, this expedited blood coagulation was toxicologically insignificant. HCT and RBC counts were significantly increased, and MCH levels were significantly decreased in the female 250 mg/kg bw/day group compared to those in the control group (*p* < 0.05, *p* < 0.01, and *p* < 0.05, respectively). As these changes were negligible and dose-independent, they were presumed not to be caused by LME administration.

β-globulin concentration in the male 500 mg/kg bw/day group was significantly higher than that in the control group (*p* < 0.05). Moreover, α1-globulin concentration in the female 1000 mg/kg bw/day group was significantly decreased compared to that in the control group (*p* < 0.05). As these changes were negligible and had no clear dose-related relationship, they were considered independent of LME administration.

**Table 4:** Hematology results of male and female rats treated with LME in the 28-day oral toxicity study.

		Male				Female			
LME (mg/kg bw/Day)		0	250	500	1000	0	250	500	1000
No. of Rats		5	5	5	5	5	5	5	5
Hematocrit	(%)	43.7 ± 1.1	44.9 ± 1.0	44.4 ± 1.1	44.9 ± 2.5	41.8 ± 1.2	43.8 ± 1.2 #	41.4 ± 0.9	42.4 ± 0.9
Hemoglobin	(g/dL)	15.4 ± 0.6	15.7 ± 0.4	15.5 ± 0.5	15.7 ± 0.9	15.2 ± 0.4	15.7 ± 0.4	14.9 ± 0.4	15.3 ± 0.3
RBC	(×10^6^/mm^3^)	7.86 ± 0.17	7.83 ± 0.39	7.85 ± 0.22	7.72 ± 0.27	7.54 ± 0.23	8.01 ± 0.22 ##	7.36 ± 0.20	7.73 ± 0.14
MCV	(µm^3^)	55.6 ± 0.6	57.5 ± 2.0	56.6 ± 2.0	58.2 ± 2.5	55.5 ± 0.9	54.7 ± 1.4	56.3 ± 0.7	54.9 ± 1.0
MCH	(pg)	19.6 ± 0.5	20.1 ± 0.7	19.7 ± 0.7	20.3 ± 0.8	20.2 ± 0.2	19.6 ± 0.3 #	20.3 ± 0.2	19.8 ± 0.5
MCHC	(%)	35.4 ± 0.5	35.0 ± 0.4	34.9 ± 0.7	34.9 ± 0.3	36.4 ± 0.3	35.8 ± 0.5	36.1 ± 0.5	36.1 ± 0.5
RETIC	(×10^9^/L)	186.1 ± 28.3	202.5 ± 30.8	210.5 ± 33.0	220.2 ± 24.4	152.5 ± 32.1	116.9 ± 16.1	159.1 ± 23.2	150.4 ± 27.6
Platelet count	(×10^3^/mm^3^)	959 ± 96	1082 ± 86	1074 ± 110	1088 ± 144	1166 ± 126	1168 ± 106	1112 ± 263	1102 ± 69
WBC	(×10^3^/mm^3^)	8.85 ± 1.21	9.80 ± 1.59	9.46 ± 3.89	10.11 ± 2.57	4.67 ± 1.81	6.61 ± 1.42	5.90 ± 1.86	5.44 ± 1.21
Neutrophil	(×10^3^/mm^3^)	1.31 ± 0.34	1.34 ± 0.20	1.85 ± 1.23	1.21 ± 0.45	0.78 ± 0.22	0.98 ± 0.68	1.10 ± 0.24	1.05 ± 0.85
Lymphocyte	(×10^3^/mm^3^)	7.17 ± 0.96	8.09 ± 1.65	7.17 ± 2.58	8.50 ± 2.24	3.65 ± 1.58	5.28 ± 0.78	4.53 ± 1.69	4.08 ± 1.27
Monocyte	(×10^3^/mm^3^)	0.23 ± 0.05	0.21 ± 0.04	0.26 ± 0.07	0.23 ± 0.06	0.12 ± 0.03	0.17 ± 0.09	0.13 ± 0.06	0.15 ± 0.05
Eosinophil	(×10^3^/mm^3^)	0.09 ± 0.02	0.09 ± 0.04	0.10 ± 0.04	0.09 ± 0.07	0.08 ± 0.03	0.14 ± 0.07	0.09 ± 0.02	0.10 ± 0.01
Basophil	(×10^3^/mm^3^)	0.01 ± 0.00	0.02 ± 0.01	0.02 ± 0.02	0.02 ± 0.01	0.00 ± 0.00	0.01 ± 0.00	0.01 ± 0.01	0.01 ± 0.01
Large unstained cell	(×10^3^/mm^3^)	0.05 ± 0.02	0.05 ± 0.02	0.07 ± 0.03	0.05 ± 0.03	0.03 ± 0.02	0.03 ± 0.01	0.05 ± 0.05	0.05 ± 0.02
PT	(s)	12.7 ± 0.4	11.3 ± 1.6	10.5 ± 0.7 **	10.2 ± 0.8 **	8.6 ± 0.3	8.6 ± 0.3	8.6 ± 0.4	8.7 ± 0.3
APTT	(s)	24.5 ± 2.2	23.5 ± 1.6	23.7 ± 1.7	23.2 ± 1.0	17.9 ± 0.5	17.5 ± 1.6	16.7 ± 1.2	17.6 ± 0.8
Fibrinogen	(mg/dL)	302 ± 31	322 ± 33	324 ± 19	314 ± 6	272 ± 31	246 ± 28	268 ± 20	269 ± 37

Data are represented as mean ± standard deviation. APTT, activated partial thromboplastin time; LME, Lemon myrtle leaf water extract; MCH, mean corpuscular hemoglobin; MCHC, mean corpuscular hemoglobin concentration; MCV, mean corpuscular volume; RBC, red blood cell count; RETIC, reticulocyte concentration; PT, prothrombin time; WBC, white blood cell count. Significantly different compared to the male 0 mg/kg bw/day group (** *p* < 0.01). Significantly different compared to the female 0 mg/kg bw/day group (# *p* < 0.05, ## *p* < 0.01).

**Table 5:** Serum biochemistry of male and female rats treated with LME in the 28-day oral toxicity study.

		Male				Female			
LME (mg/kg bw/Day)		0	250	500	1000	0	250	500	1000
No. of Rats		5	5	5	5	5	5	5	5
Glucose	(mg/dL)	147 ± 14	154 ± 7	144 ± 12	153 ± 17	123 ± 11	121 ± 19	129 ± 24	105 ± 9
Triglyceride	(mg/dL)	41 ± 27	67 ± 27	45 ± 8	63 ± 18	21 ± 3	21 ± 16	25 ± 12	23 ± 7
Total cholesterol	(mg/dL)	60 ± 13	63 ± 11	58 ± 8	58 ± 14	65 ± 14	62 ± 10	68 ± 16	56 ± 16
Blood urea nitrogen	(mg/dL)	13.2 ± 1.6	12.6 ± 0.9	13.3 ± 1.3	12.3 ± 1.3	15.0 ± 1.8	19.3 ± 5.3	15.8 ± 1.7	13.8 ± 2.9
Creatinine	(mg/dL)	0.28 ± 0.02	0.27 ± 0.01	0.30 ± 0.07	0.25 ± 0.03	0.31 ± 0.05	0.35 ± 0.08	0.34 ± 0.01	0.28 ± 0.06
Total bilirubin	(mg/dL)	0.05 ± 0.01	0.05 ± 0.01	0.06 ± 0.01	0.05 ± 0.02	0.06 ± 0.01	0.06 ± 0.02	0.07 ± 0.02	0.07 ± 0.01
Aspartate aminotransferase	(U/L)	74 ± 7	70 ± 9	67 ± 11	70 ± 12	73 ± 15	64 ± 5	67 ± 10	68 ± 8
Alanine aminotransferase	(U/L)	30 ± 2	26 ± 5	26 ± 5	47 ± 49	21 ± 1	21 ± 3	22 ± 3	20 ± 2
Alkaline phosphatase	(U/L)	631 ± 98	563 ± 141	660 ± 100	571 ± 71	390 ± 121	318 ± 90	382 ± 118	282 ± 50
γ-GPT	(U/L)	0.6 ± 0.2	0.4 ± 0.1	0.6 ± 0.4	0.5 ± 0.2	0.7 ± 0.3	0.7 ± 0.3	0.8 ± 0.3	0.9 ± 0.3
Calcium	(mg/dL)	9.36 ± 0.24	9.57 ± 0.44	9.36 ± 0.29	9.81 ± 0.16	9.39 ± 0.22	9.67 ± 0.19	9.79 ± 0.38	9.54 ± 0.34
Inorganic phosphorus	(mg/dL)	7.34 ± 0.36	7.71 ± 0.41	7.53 ± 0.52	7.32 ± 0.37	6.06 ± 0.41	6.50 ± 0.64	6.04 ± 0.74	6.39 ± 0.43
Total protein	(g/dL)	5.37 ± 0.27	5.53 ± 0.19	5.43 ± 0.11	5.48 ± 0.12	5.92 ± 0.39	5.70 ± 0.31	5.88 ± 0.31	5.65 ± 0.20
Albumin	(g/dL)	2.87 ± 0.15	2.88 ± 0.08	2.82 ± 0.09	2.82 ± 0.13	3.30 ± 0.19	3.25 ± 0.24	3.36 ± 0.32	3.22 ± 0.13
α1-globulin	(g/dL)	1.18 ± 0.10	1.24 ± 0.14	1.17 ± 0.16	1.23 ± 0.14	1.14 ± 0.19	1.05 ± 0.12	1.06 ± 0.08	0.92 ± 0.12 #
α2-globulin	(g/dL)	0.35 ± 0.03	0.39 ± 0.03	0.38 ± 0.03	0.39 ± 0.02	0.38 ± 0.05	0.43 ± 0.05	0.42 ± 0.07	0.46 ± 0.08
β-globulin	(g/dL)	0.77 ± 0.05	0.83 ± 0.04	0.89 ± 0.09 *	0.86 ± 0.04	0.82 ± 0.06	0.75 ± 0.03	0.81 ± 0.06	0.81 ± 0.08
γ-globulin	(g/dL)	0.19 ± 0.10	0.20 ± 0.07	0.17 ± 0.05	0.18 ± 0.05	0.28 ± 0.08	0.22 ± 0.07	0.23 ± 0.11	0.25 ± 0.01
A/G		1.16 ± 0.12	1.09 ± 0.02	1.08 ± 0.07	1.07 ± 0.11	1.26 ± 0.11	1.33 ± 0.11	1.35 ± 0.18	1.34 ± 0.11
Sodium	(mmol/L)	143.5 ± 0.4	143.5 ± 0.9	143.6 ± 0.2	143.8 ± 0.6	143.4 ± 0.7	143.2 ± 1.5	143.8 ± 1.3	144.2 ± 1.2
Potassium	(mmol/L)	4.58 ± 0.11	4.90 ± 0.38	4.70 ± 0.31	4.79 ± 0.28	4.64 ± 0.33	4.40 ± 0.27	4.38 ± 0.15	4.54 ± 0.39
Chloride	(mmol/L)	106.3 ± 0.7	105.9 ± 1.3	106.4 ± 0.4	105.4 ± 1.0	108.6 ± 1.8	107.8 ± 1.9	107.7 ± 1.6	109.1 ± 2.4

Data are represented as mean ± standard deviation. A/G, albumin-to-globulin ratio; γ-GPT, γ-glutamyl transpeptidase activity; LME, Lemon myrtle leaf water extract. Significantly different compared to the male 0 mg/kg bw/day group (* *p* < 0.05). Significantly different compared to the female 0 mg/kg bw/day group (# *p* < 0.05).

#### 3.2.4. Organ Weights

The organ weight data are presented in Table 6. In the liver, the absolute and relative weights were significantly increased in males treated with the 1000 mg/kg bw/day group compared with those in the control group (*p* < 0.01 and *p* < 0.05, respectively). The absolute adrenal gland weight was significantly higher in the male 250 and 1000 mg/kg bw/day groups than in the control group (*p* < 0.01). The absolute lung and liver weights were significantly increased in the male 500 mg/kg bw/day group, while the absolute heart weight was significantly increased in the male 1000 mg/kg bw/day group, compared to those in the control group (*p* < 0.05). In the female 250 mg/kg bw/day group, the absolute pituitary weight was significantly lower than that in the control group (*p* < 0.05).

**Table 6:** Organ weights of male and female rats treated with LME in the 28-day oral toxicity study.

		Male				Female			
LME (mg/kg bw/Day)		0	250	500	1000	0	250	500	1000
No. of Rats		5	5	5	5	5	5	5	5
Final body weight	(g)	340 ± 21	363 ± 12	362 ± 23	368 ± 27	229 ± 20	216 ± 16	237 ± 16	211 ± 8
Brain	Absolute (g)	1.95 ± 0.08	1.98 ± 0.10	2.10 ± 0.12	1.99 ± 0.07	1.89 ± 0.04	1.82 ± 0.09	1.88 ± 0.09	1.85 ± 0.12
	Relative (%)	0.575 ± 0.023	0.548 ± 0.037	0.581 ± 0.061	0.542 ± 0.036	0.832 ± 0.064	0.845 ± 0.060	0.795 ± 0.063	0.877 ± 0.049
Heart	Absolute (g)	1.10 ± 0.03	1.20 ± 0.09	1.18 ± 0.07	1.22 ± 0.05 *	0.80 ± 0.06	0.76 ± 0.11	0.79 ± 0.05	0.77 ± 0.08
	Relative (%)	0.323 ± 0.012	0.330 ± 0.026	0.326 ± 0.020	0.334 ± 0.019	0.352 ± 0.019	0.352 ± 0.031	0.334 ± 0.013	0.363 ± 0.029
Lung	Absolute (g)	1.18 ± 0.13	1.30 ± 0.03	1.35 ± 0.08 *	1.28 ± 0.08	1.01 ± 0.11	1.00 ± 0.06	1.06 ± 0.08	0.99 ± 0.06
	Relative (%)	0.347 ± 0.028	0.360 ± 0.014	0.374 ± 0.013	0.348 ± 0.016	0.440 ± 0.032	0.466 ± 0.041	0.449 ± 0.032	0.471 ± 0.019
Liver	Absolute (g)	8.98 ± 0.86	10.09 ± 0.62	10.42 ± 0.39 *	10.86 ± 1.28 **	6.22 ± 0.48	5.77 ± 0.40	6.60 ± 0.86	5.75 ± 0.28
	Relative (%)	2.637 ± 0.149	2.784 ± 0.174	2.881 ± 0.108	2.945 ± 0.161 *	2.728 ± 0.194	2.671 ± 0.117	2.780 ± 0.217	2.723 ± 0.080
Kidneys	Absolute (g)	2.46 ± 0.15	2.53 ± 0.15	2.68 ± 0.29	2.76 ± 0.26	1.76 ± 0.11	1.60 ± 0.18	1.65 ± 0.14	1.68 ± 0.15
	Relative (%)	0.723 ± 0.027	0.697 ± 0.048	0.740 ± 0.070	0.749 ± 0.051	0.772 ± 0.063	0.741 ± 0.050	0.698 ± 0.026	0.797 ± 0.050
Spleen	Absolute (g)	0.68 ± 0.16	0.62 ± 0.14	0.69 ± 0.07	0.68 ± 0.06	0.47 ± 0.10	0.43 ± 0.06	0.49 ± 0.04	0.41 ± 0.04
	Relative (%)	0.199 ± 0.046	0.171 ± 0.046	0.192 ± 0.028	0.184 ± 0.010	0.204 ± 0.039	0.199 ± 0.033	0.206 ± 0.024	0.193 ± 0.026
Thymus	Absolute (mg)	514 ± 128	495 ± 78	479 ± 70	484 ± 81	430 ± 108	478 ± 123	462 ± 104	362 ± 41
	Relative (%)	0.151 ± 0.034	0.136 ± 0.020	0.133 ± 0.021	0.131 ± 0.017	0.191 ± 0.058	0.220 ± 0.049	0.198 ± 0.055	0.172 ± 0.022
Adrenal glands	Absolute (mg)	45 ± 5	58 ± 7 **	55 ± 7	59 ± 6 **	64 ± 10	58 ± 5	64 ± 4	62 ± 3
	Relative (%)	0.013 ± 0.002	0.016 ± 0.002	0.015 ± 0.002	0.016 ± 0.002	0.028 ± 0.004	0.027 ± 0.004	0.027 ± 0.003	0.030 ± 0.002
Thyroid glands	Absolute (mg)	22 ± 4	21 ± 6	22 ± 4	23 ± 4	20 ± 3	19 ± 5	18 ± 5	20 ± 4
	Relative (%)	0.006 ± 0.001	0.006 ± 0.002	0.006 ± 0.001	0.006 ± 0.001	0.009 ± 0.001	0.009 ± 0.003	0.007 ± 0.002	0.010 ± 0.002
Pituitary	Absolute (mg)	9 ± 2	11 ± 1	11 ± 0	10 ± 1	14 ± 2	10 ± 2 #	12 ± 2	11 ± 2
	Relative (%)	0.003 ± 0.001	0.003 ± 0.000	0.003 ± 0.000	0.003 ± 0.000	0.006 ± 0.001	0.005 ± 0.001	0.005 ± 0.001	0.005 ± 0.001
Salivary glands	Absolute (g)	0.60 ± 0.06	0.62 ± 0.07	0.68 ± 0.07	0.60 ± 0.04	0.42 ± 0.02	0.37 ± 0.03	0.43 ± 0.04	0.39 ± 0.04
	Relative (%)	0.175 ± 0.010	0.172 ± 0.018	0.187 ± 0.015	0.163 ± 0.016	0.186 ± 0.020	0.174 ± 0.019	0.181 ± 0.027	0.183 ± 0.021
Testes	Absolute (g)	3.16 ± 0.37	3.09 ± 0.14	3.21 ± 0.23	3.30 ± 0.17	-	-	-	-
	Relative (%)	0.934 ± 0.146	0.852 ± 0.033	0.888 ± 0.077	0.902 ± 0.111	-	-	-	-
Epididymides	Absolute (mg)	814 ± 76	798 ± 52	821 ± 28	846 ± 44	-	-	-	-
	Relative (%)	0.240 ± 0.024	0.220 ± 0.011	0.227 ± 0.015	0.231 ± 0.020	-	-	-	-
Prostate	Absolute (mg)	1086 ± 78	1088 ± 128	1235 ± 142	1111 ± 162	-	-	-	-
	Relative (%)	0.320 ± 0.028	0.300 ± 0.029	0.341 ± 0.029	0.304 ± 0.058	-	-	-	-
Seminal vesicle	Absolute (mg)	1066 ± 101	1097 ± 140	1136 ± 121	1011 ± 135	-	-	-	-
	Relative (%)	0.313 ± 0.028	0.302 ± 0.029	0.313 ± 0.026	0.276 ± 0.047	-	-	-	-
Ovaries	Absolute (mg)	-	-	-	-	84 ± 10	73 ± 9	82 ± 13	84 ± 23
	Relative (%)	-	-	-	-	0.037 ± 0.003	0.034 ± 0.006	0.035 ± 0.005	0.040 ± 0.011
Uterus	Absolute (mg)	-	-	-	-	508 ± 112	405 ± 51	525 ± 150	471 ± 102
	Relative (%)	-	-	-	-	0.221 ± 0.039	0.187 ± 0.020	0.225 ± 0.076	0.224 ± 0.049

Data are represented as mean ± standard deviation. LME, Lemon myrtle leaf water extract. -, Not applicable. Significantly different compared to the male 0 mg/kg bw/day group (* *p* < 0.05, ** *p* < 0.01). Significantly different compared to the female 0 mg/kg bw/day group (# *p* < 0.05).

#### 3.2.5. Macroscopic Examination and Histopathology

Gross necropsy findings and histopathological data are presented in Table S3 and Table 7, respectively. Gross necropsy revealed a nodule in the spleen of 1 male rat in the 250 mg/kg bw/day group. In the lungs, brown patches and browning were observed in one male rat each in the 250 and 500 mg/kg bw/day groups, respectively. Hepatodiaphragmatic nodules and white patches were observed in the liver of 1 male rat in the 250 mg/kg bw/day group. Kidney cysts were observed in 1 male rat each in the control and 250 mg/kg bw/day groups, and focal depression was observed in 1, 2, 1, and 1 male rat in the control, 250, 500, and 1000 mg/kg bw/day groups, respectively. In female rats, hepatodiaphragmatic nodules in the liver and focal depression in the kidneys were observed in 1 and 2 rat in the 500 mg/kg bw/day group, respectively. All findings were considered spontaneous as they occurred singly or sporadically in only a few cases.

Histopathologically, hyaline droplets in the kidneys were observed in 1 (slight), 3 (slight), 5 (slight: 1 and moderate: 4), and 5 (moderate) male rats in the control, 250, 500, and 1000 mg/kg bw/day groups, respectively. All other findings were considered spontaneous based on the type, number, extent, and morphological characteristics of the lesions.

**Table 7:** Histopathological findings of male and female rats treated with LME in the 28-day oral toxicity study.

		Male				Female			
LME (mg/kg bw/Day)		0	250	500	1000	0	250	500	1000
Organ/Finding	Grade								
Heart		<5> ^a^	<0>	<0>	<5>	<5>	<0>	<0>	<5>
Cardiomyopathy	-	3	NA	NA	5	5	NA	NA	5
	+	2	NA	NA	0	0	NA	NA	0
Lung		<5>	<0>	<0>	<5>	<5>	<0>	<0>	<5>
Metaplasia, osseous	-	5	NA	NA	4	5	NA	NA	5
	+	0	NA	NA	1	0	NA	NA	0
Aggregation, macrophage	-	3	NA	NA	4	5	NA	NA	5
	+	2	NA	NA	1	0	NA	NA	0
Inflammatory change	-	4	NA	NA	5	4	NA	NA	5
	+	1	NA	NA	0	1	NA	NA	0
Nasal cavity		<5>	<0>	<0>	<5>	<5>	<0>	<0>	<5>
Mineralization	-	1	NA	NA	0	0	NA	NA	1
	+	4	NA	NA	5	5	NA	NA	4
Pancreas (exocrine)		<5>	<0>	<0>	<5>	<5>	<0>	<0>	<5>
Inflammatory change	-	5	NA	NA	4	5	NA	NA	5
	+	0	NA	NA	1	0	NA	NA	0
Liver		<5>	<0>	<0>	<5>	<5>	<0>	<0>	<5>
Fatty change, hepatocyte	-	5	NA	NA	5	5	NA	NA	4
	+	0	NA	NA	0	0	NA	NA	1
Microgranuloma	-	0	NA	NA	0	1	NA	NA	0
	+	5	NA	NA	5	4	NA	NA	5
Kidney		<5> ^a^	<5>	<5>	<5>	<5>	<0>	<0>	<5>
Cyst	-	4	4	5	5	5	NA	NA	5
	+	1	1	0	0	0	NA	NA	0
Hyaline droplet	-	4	2	0	0	5	NA	NA	5
	+	1	3	1	0	0	NA	NA	0
	++	0	0	4	5	0	NA	NA	0
Mineralization	-	5	5	4	4	5	NA	NA	4
	+	0	0	1	1	0	NA	NA	1
Regeneration, tubule	-	1	3	2	3	3	NA	NA	5
	+	4	2	3	2	2	NA	NA	0
Inflammatory change	-	4	5	3	5	5	NA	NA	5
	+	1	0	2	0	0	NA	NA	0
Epididymis		<5>	<0>	<0>	<5>	<NA>	<NA>	<NA>	<NA>
Infiltration, inflammatory cell	-	4	NA	NA	3	NA	NA	NA	NA
	+	1	NA	NA	2	NA	NA	NA	NA
Prostate		<5>	<0>	<0>	<5>	<NA>	<NA>	<NA>	<NA>
Inflammatory change	-	1	NA	NA	1	NA	NA	NA	NA
	+	4	NA	NA	4	NA	NA	NA	NA

LME, Lemon myrtle leaf water extract. ^a^ Numbers in angle brackets are for animals examined microscopically. Grade: -; Normal, +; Slight, ++; Moderate, NA; Not applicable.

## 4. Discussion

To assess the safety of LME, we evaluated genotoxicity using the Ames test, in vivo mammalian erythrocyte micronucleus test, and in vivo alkaline comet assay and conducted a 28-day repeated-dose oral toxicity study. In the Ames test, LME was positive in the TA98 strain with or without S9, but negative in the mammalian erythrocyte micronucleus test and the in vivo alkaline comet assay. The results of the 28-day repeated oral dose toxicity study revealed no findings of toxicological significance due to LME administration.

Among the major compounds in LME, namely gallic acid, myricitrin, hyperin, quercitrin, and casuarinin, the Ames test using the pre-incubation method revealed negative results for gallic acid in the TA98 and TA100 strains (highest concentration of 1000 µg/plate) [25,26]; for myricitrin in the TA97a, TA98, TA100, TA1535, and WP2*uvrA* strains (highest concentration of 5000 µg/plate) [27]; and for hyperin in the TA97, TA98, TA100, and TA102 strains (highest concentration of 2000 µg/plate), both in the absence and presence of S9 [28]. In contrast, for quercitrin, the Ames test yields positive results in the presence of S9 in the TA98 strain [29]. However, unlike the three substances mentioned above (using the two-fold method or statistical analysis), a positive criterion of more than 0.10 revertants/nmol was used, under which quercitrin was judged positive with 0.43 revertants/nmol. In addition, the number of revertant colonies in the TA98 strain under the presence of S9 increased more than two-fold at the lowest dose of 500 µg/plate compared to the negative control. To the best of our knowledge, there are no reported Ames test analyses for casuarinin. At the highest concentration of 5000 µg/plate used in this Ames test, the amounts of gallic acid, myricitrin, hyperin, quercitrin, and casuarinin were 12.5, 63.0, 95.5, 194.0, and 80.5 µg/plate, respectively. Notably, gallic acid, myricitrin, and hyperin have tested negative in previous studies, and the concentrations used in the present study were lower than the highest concentrations reported in these previous studies. Therefore, it is unlikely that these substances are responsible for the positive results observed in the TA98 strain in the absence or presence of S9 in this Ames test. Although the content of quercitrin in this study was lower than that previously reported, it is possible that it exhibited a positive result in the TA98 strain under the presence of S9. In addition, flavonoids modulate the activities of enzymes involved in the biotransformation of procarcinogens, altering their biological activity. Flavonoids may also exhibit genotoxic activity by yielding reactive intermediates such as free radical species [30]. Among the major LME compounds, myricitrin, hyperin, and quercitrin are flavonoids. Therefore, the positive result observed for LME in the Ames test in the present study may be attributed to quercitrin or other unidentified compounds contained in LME.

In the present study, the Ames test was conducted up to the maximum concentration of 5000 µg/plate or until precipitation and growth inhibition were observed, in strict accordance with OECD Test Guideline 471 [20]. This protocol ensured that the test strains were subjected to a sufficient concentration of LME to thoroughly evaluate its mutagenic potential. Although LME yielded positive results in the TA98 strain regardless of the presence or absence of S9, other strains designed to detect frameshift mutations, such as TA1537, remained negative. This strain-specific sensitivity is primarily attributed to the presence of the pKM101 plasmid in TA98, which is absent in TA1537. The pKM101 plasmid enhances the error-prone DNA repair pathway (translesion synthesis or “damage-bypass” replication), thereby increasing the detection sensitivity for mutations induced by specific DNA-reactive agents [31]. Furthermore, the *uvr*B deletion mutation in these strains abolishes accurate nucleotide excision repair (NER) and the *rfa* mutation increases permeability to bulky chemicals, sensitizing the bacterial system to potential DNA damage. The positivity in the TA98 strain indicates the induction of frameshift mutations, likely involving the formation of DNA adducts.

Bulky DNA adducts are among the types of DNA damage normally repaired by NER [32], and substances that form DNA adducts—such as benzo[a]pyrene, AAF, and AF—are known to induce frameshift mutations [33,34,35]. Therefore, a subsequent in vivo mammalian alkaline comet assay was performed to facilitate the detection of the DNA damage associated with adduct formation. The results of this assay were negative, as no significant differences were observed in % tail DNA or the frequency of hedgehogs in the glandular stomach or liver cells between the LME-treated and negative control groups.

Furthermore, the mammalian erythrocyte micronucleus test revealed no significant changes in PCE% incidence or MNPCE% incidence in mice treated with LME, confirming the absence of myelotoxicity. Although DNA damage and chromosomal aberrations are generally considered to precede gene mutations, neither DNA damage nor chromosomal aberrations were observed in the present study. These findings suggest that the mutagenic metabolites or mechanisms observed in vitro are likely detoxified or neutralized under physiological conditions in vivo. While the in vitro positivity indicates potential for DNA reactivity under specific conditions, the comprehensive in vivo data provide reassuring evidence that LME does not pose a substantial genotoxic risk at practical oral exposure levels. Consequently, within the scope of these regulatory-compliant studies, LME can be considered to be of a low concern for genotoxicity in a clinical or dietary context.

In the 28-day rat oral toxicity study, hyaline droplets were observed in the proximal tubules of the kidneys in male rats among all treatment groups, with a dose-dependent increase in the number of cases and an enhancement in the degree of findings, which was considered to be an effect of LME. In adult male rats, α2-microglobulin binds to various chemicals, resulting in increased hyaline droplets in the proximal tubular epithelial cells and damage to the tubular epithelium. However, this result cannot be extrapolated to humans because they lack α2-microglobulin [36]. In the present study, hyaline droplets in the proximal tubules were only observed in males, correlating these changes with the deposition of α2-microglobulin. However, no toxic effects were observed in the 1000 mg/kg bw/day group, and no damage to the tubular epithelial cells was detected. In contrast, significant changes in organ weights were observed in the LME groups. However, these changes were not attributed to LME administration, as no corresponding histopathological findings were observed. In addition, no changes related to LME administration in the general conditions, food consumption, urinalysis, or ophthalmologic examination results were observed. Based on these results, the NOAEL of LME was considered to be 1000 mg/kg bw/day for males and females, under the conditions of the present study. This NOAEL corresponds to an exposure margin of approximately 387-fold when calculated from a daily dose of 155 mg as LME used in the clinical trial [14], assuming a human body weight of 60 kg.

## 5. Conclusions

The NOAEL of LME was 1000 mg/kg bw/day for male and female rats under the conditions of the present 28-day rat oral toxicity study. Although the observed Ames test results were positive, the in vivo mammalian erythrocyte micronucleus test and in vivo mammalian alkaline comet assay did not reliably indicate in vivo genotoxicity attributable to LME administration. Despite its genotoxicity in vitro, LME did not exhibit genotoxicity in vivo, and no signs of toxicity were observed in the general toxicity study. While LME exhibited a positive result in the Ames test using the TA98 strain, the causative compound remains unidentified and requires further investigation. Overall, these results suggest that LME has a sufficient safety margin (approximately a 387-fold exposure margin) relative to the clinical dose (155 mg/day as LME) and does not pose a substantive genotoxic risk at practical oral exposure levels.

## Data Availability

The original contributions presented in this study are included in the article/Supplementary Material. Further inquiries can be directed to the corresponding author.

## Supplementary Materials

The following supporting information can be downloaded at: https://www.mdpi.com/article/10.3390/toxics14030213/s1, Table S1: Ophthalmology of male and female rats treated with LME in the 28-day oral toxicity study; Table S2: Urinalysis of male and female rats treated with LME in the 28-day oral toxicity study; Table S3: Gross necropsy findings of male and female rats treated with LME in the 28-day oral toxicity study; Figure S1: Microscopic images in the liver in the in vivo mammalian alkaline comet assay; Figure S2: Changes in the body weight of rats; Figure S3: Body weight gains in rats; Figure S4: Changes in food consumption among rats.
