# Supplementary material for: Safety Assessment of Lemon Myrtle (Backhousia citriodora) Extract: 28-Day Oral Toxicity Study in Rats and In Vitro and In Vivo Genotoxicity Studies
Source: Toxics. 2026 Feb 28;14(3):213. doi: 10.3390/toxics14030213 (PMC13029901; doi:10.3390/toxics14030213)
Supplement: Supplementary file 1 [file toxics-14-00213-s001.zip › toxics-4131252-supplementary.pdf]

Supplementary Materials

Table S1. Ophthalmology of male and female rats treated with LME in the 28-day oral toxicity study.

		**Male**		**Female**	
**LME (mg/kg bw/Day)**		**0**	**1000**	**0**	**1000**
**No. of Rats**		**5**	**5**	**5**	**5**
＜Day -2＞					
Anterior part (Conjunctiva, Sclera, Cornea, Iris)					
Corneal opacity	-	3	2	4	4
	+	2	3	1	1
Optic media (Posterior chamber, Lens, Corpus vitreum)					
	-	5	5	5	5
Fundus oculi					
Chorioretinal atrophy	-	5	5	5	5
	+	0	0	0	0
＜Day 27＞					
Anterior part (Conjunctiva, Sclera, Cornea, Iris)					
Corneal opacity	-	2	2	3	4
	+	3	3	2	1
Optic media (Posterior chamber, Lens, Corpus vitreum)					
	-	5	5	5	5
Fundus oculi					
Chorioretinal atrophy	-	5	5	4	5
	+	0	0	1	0

LME, Lemon Myrtle leaf water extract.

-: Normal, +: presence of finding

Table S2. Urinalysis of male and female rats treated with LME in the 28-day oral toxicity study.

		Male				Female			
**LME (mg/kg bw/Day)**		**0**	**250**	**500**	**1000**	**0**	**250**	**500**	**1000**
**No. of Rats**		**5**	**5**	**5**	**5**	**5**	**5**	**5**	**5**
< Day -4 and -3>									
Color	Slight yellow	5	4	4	5	5	5	5	5
	Yellow-brown	0	1	1	0	0	0	0	0
pH	7.5	0	0	0	0	0	0	0	0
	8.0	0	0	0	0	0	0	0	0
	8.5	3	0	3	1	4	2	2	2
	≥ 9.0	2	5	2	4	1	3	3	3
Occult blood	-	5	4	5	5	5	4	5	4
	+/-	0	1	0	0	0	1	0	1
	1+	0	0	0	0	0	0	0	0
	2+	0	0	0	0	0	0	0	0
	3+	0	0	0	0	0	0	0	0
Ketone bodies	-	3	2	2	3	3	3	4	3
	+/-	2	1	3	2	2	2	1	2
	1+	0	2	0	0	0	0	0	0
	2+	0	0	0	0	0	0	0	0
	3+	0	0	0	0	0	0	0	0
	4+	0	0	0	0	0	0	0	0
Glucose	-	5	5	5	5	5	5	5	5
	0.1 g/dL	0	0	0	0	0	0	0	0
	0.25 g/dL	0	0	0	0	0	0	0	0
	0.5 g/dL	0	0	0	0	0	0	0	0
	≥ 1.0 g/dL	0	0	0	0	0	0	0	0
Protein	-	2	1	0	0	2	2	2	3
	+/-	1	0	3	3	2	3	1	2
	30 mg/dL	1	3	2	2	1	0	2	0
	100 mg/dL	1	1	0	0	0	0	0	0
	≥ 300 mg/dL	0	0	0	0	0	0	0	0
Bilirubin	-	5	4	4	5	4	5	5	5
	1+	0	1	1	0	1	0	0	0
	2+	0	0	0	0	0	0	0	0
	3+	0	0	0	0	0	0	0	0

Table S2. *Cont.*

LME (mg/kg bw/Day)		Male 0	Male 250	Male 500	Male 1000	Female 0	Female 250	Female 500	Female 1000
**No. of Rats**		**5**	**5**	**5**	**5**	**5**	**5**	**5**	**5**
< Day -4 and -3>									
Urobilinogen	0.1 E.U./dL	3	3	3	4	4	5	3	4
	1.0 E.U./dL	2	2	2	1	1	0	2	1
	2.0 E.U./dL	0	0	0	0	0	0	0	0
	4.0 E.U./dL	0	0	0	0	0	0	0	0
	8.0 E.U./dL	0	0	0	0	0	0	0	0
	≥ 12.0 E.U./dL	0	0	0	0	0	0	0	0
Erythrocytes	-	5	5	5	5	5	5	5	5
	1+	0	0	0	0	0	0	0	0
	2+	0	0	0	0	0	0	0	0
	3+	0	0	0	0	0	0	0	0
Leukocytes	-	5	5	5	5	5	5	5	5
	1+	0	0	0	0	0	0	0	0
	2+	0	0	0	0	0	0	0	0
	3+	0	0	0	0	0	0	0	0
Squamous cells	-	5	5	5	5	5	5	5	5
	1+	0	0	0	0	0	0	0	0
	2+	0	0	0	0	0	0	0	0
	3+	0	0	0	0	0	0	0	0
Transitional epithelial cells	-	5	5	5	5	5	5	5	5
	1+	0	0	0	0	0	0	0	0
	2+	0	0	0	0	0	0	0	0
	3+	0	0	0	0	0	0	0	0
Renal tubular epithelial cells	-	5	5	5	5	5	5	5	5
	1+	0	0	0	0	0	0	0	0
	2+	0	0	0	0	0	0	0	0
	3+	0	0	0	0	0	0	0	0

Table S2. *Cont.*

LME (mg/kg bw/Day)		Male				Female			
		**0**	**250**	**500**	**1000**	**0**	**250**	**500**	**1000**
**No. of Rats**		**5**	**5**	**5**	**5**	**5**	**5**	**5**	**5**
< Day 26 and 27>									
Color	Slight yellow	5	5	5	5	5	5	5	5
	Yellow-brown	0	0	0	0	0	0	0	0
pH	7.5	0	0	0	0	0	0	0	1
	8.0	0	0	0	0	1	0	1	1
	8.5	4	3	2	0	4	1	3	0
	≥ 9.0	1	2	3	5	0	4	1	3
Occult blood	-	4	3	5	5	3	5	5	5
	+/-	1	1	0	0	2	0	0	0
	1+	0	1	0	0	0	0	0	0
	2+	0	0	0	0	0	0	0	0
	3+	0	0	0	0	0	0	0	0
Ketone bodies	-	4	2	2	1	5	4	3	4
	+/-	1	3	2	4	0	1	2	0
	1+	0	0	1	0	0	0	0	1
	2+	0	0	0	0	0	0	0	0
	3+	0	0	0	0	0	0	0	0
	4+	0	0	0	0	0	0	0	0
Glucose	-	5	5	5	5	5	5	5	5
	0.1 g/dL	0	0	0	0	0	0	0	0
	0.25 g/dL	0	0	0	0	0	0	0	0
	0.5 g/dL	0	0	0	0	0	0	0	0
	≥ 1.0 g/dL	0	0	0	0	0	0	0	0
Protein	-	4	4	0	1	5	4	1	4
	+/-	1	0	2	3	0	0	3	0
	30 mg/dL	0	1	3	1	0	1	1	0
	100 mg/dL	0	0	0	0	0	0	0	1
	≥ 300 mg/dL	0	0	0	0	0	0	0	0
Bilirubin	-	5	5	5	5	5	5	5	4
	1+	0	0	0	0	0	0	0	1
	2+	0	0	0	0	0	0	0	0
	3+	0	0	0	0	0	0	0	0

Table S2. *Cont.*

		Male				Female			
**LME (mg/kg bw/Day)**		**0**	**250**	**500**	**1000**	**0**	**250**	**500**	**1000**
**No. of Rats**		**5**	**5**	**5**	**5**	**5**	**5**	**5**	**5**
< Day 26 and 27>									
Urobilinogen	0.1 E.U./dL	5	5	5	5	5	4	4	4
	1.0 E.U./dL	0	0	0	0	0	1	1	1
	2.0 E.U./dL	0	0	0	0	0	0	0	0
	4.0 E.U./dL	0	0	0	0	0	0	0	0
	8.0 E.U./dL	0	0	0	0	0	0	0	0
	≥ 12.0 E.U./dL	0	0	0	0	0	0	0	0
Erythrocytes	-	5	5	5	5	5	5	5	5
	1+	0	0	0	0	0	0	0	0
	2+	0	0	0	0	0	0	0	0
	3+	0	0	0	0	0	0	0	0
Leukocytes	-	5	5	5	5	4	5	4	4
	1+	0	0	0	0	1	0	1	1
	2+	0	0	0	0	0	0	0	0
	3+	0	0	0	0	0	0	0	0
Squamous cells	-	5	5	5	5	5	5	5	5
	1+	0	0	0	0	0	0	0	0
	2+	0	0	0	0	0	0	0	0
	3+	0	0	0	0	0	0	0	0
Transitional epithelial	-	5	5	5	5	5	5	5	5
cells	1+	0	0	0	0	0	0	0	0
	2+	0	0	0	0	0	0	0	0
	3+	0	0	0	0	0	0	0	0
Renal tubular epithelial	-	5	5	5	5	5	5	5	5
cells	1+	0	0	0	0	0	0	0	0
	2+	0	0	0	0	0	0	0	0
	3+	0	0	0	0	0	0	0	0

LME, Lemon Myrtle leaf water extract.

Table S2. *Cont.*

LME (mg/kg bw/Day)		Male 0	Male 250	Male 500	Male 1000	Female 0	Female 250	Female 500	Female 1000
**No. of Rats**		**5**	**5**	**5**	**5**	**5**	**5**	**5**	**5**
< Day -4 and -3>									
Volume	(mL)	7.4 ± 1.5	6.4 ± 1.2	7.7 ± 1.2	8.3 ± 2.0	8.2 ± 2.3	7.2 ± 2.3	8.8 ± 2.9	6.0 ± 1.8
Osmotic pressure	(mOsm/kg)	1778 ± 260	1914 ± 435	1763 ± 326	1547 ± 467	1474 ± 229	1547 ± 246	1524 ± 320	1690 ± 203
Sodium	(mmol/L)	179.3 ± 34.6	184.6 ± 58.4	154.4 ± 24.7	149.9 ± 45.5	118.4 ± 14.3	143.8 ± 21.8	129.9 ± 26.6	144.9 ± 28.2
	(mmol/day)	1.29 ± 0.07	1.14 ± 0.19	1.16 ± 0.05	1.17 ± 0.17	0.95 ± 0.22	1.01 ± 0.22	1.10 ± 0.22	0.83 ± 0.16
Potassium	(mmol/L)	322.1 ± 64.5	357.0 ± 86.4	323.3 ± 74.1	277.4 ± 90.7	235.7 ± 49.7	254.0 ± 40.2	250.9 ± 57.0	279.4 ± 33.6
	(mmol/day)	2.33 ± 0.21	2.21 ± 0.22	2.41 ± 0.27	2.15 ± 0.40	1.87 ± 0.42	1.78 ± 0.40	2.08 ± 0.16	1.63 ± 0.37
Chloride	(mmol/L)	245.4 ± 50.2	272.6 ± 68.5	231.0 ± 42.9	208.8 ± 67.7	181.1 ± 31.8	199.0 ± 34.5	192.1 ± 34.2	208.1 ± 23.8
	(mmol/day)	1.77 ± 0.08	1.69 ± 0.19	1.73 ± 0.14	1.62 ± 0.28	1.45 ± 0.31	1.38 ± 0.28	1.61 ± 0.22	1.22 ± 0.30
＜Day 26 and 27＞									
Volume	(mL)	15.7 ± 4.5	14.3 ± 4.0	18.0 ± 6.2	21.4 ± 7.7	12.2 ± 7.3	8.7 ± 1.8	11.7 ± 2.4	9.4 ± 5.8
Osmotic pressure	(mOsm/kg)	1353 ± 342	1586 ± 402	1322 ± 277	999 ± 417	1477 ± 399	1612 ± 322	1652 ± 256	1769 ± 832
Sodium	(mmol/L)	96.4 ± 18.8	121.6 ± 28.5	88.6 ± 13.9	66.6 ± 42.1	103.2 ± 30.2	115.2 ± 24.8	114.1 ± 14.3	125.8 ± 63.0
	(mmol/day)	1.45 ± 0.29	1.67 ± 0.29	1.56 ± 0.44	1.20 ± 0.37	1.13 ± 0.38	0.97 ± 0.18	1.34 ± 0.33	0.91 ± 0.17
Potassium	(mmol/L)	197.1 ± 54.8	246.4 ± 69.3	203.6 ± 42.7	168.5 ± 68.7	194.2 ± 54.3	218.0 ± 44.9	237.0 ± 37.3	255.4 ± 122.9
	(mmol/day)	2.90 ± 0.38	3.33 ± 0.24	3.46 ± 0.54	3.22 ± 0.55	2.11 ± 0.64	1.84 ± 0.27	2.73 ± 0.42	1.89 ± 0.44
Chloride	(mmol/L)	136.9 ± 39.2	173.7 ± 50.5	143.3 ± 27.3	114.2 ± 64.6	147.6 ± 42.7	161.5 ± 41.7	179.7 ± 26.3	199.2 ± 100.8
	(mmol/day)	2.02 ± 0.29	2.35 ± 0.32	2.47 ± 0.55	2.11 ± 0.69	1.64 ± 0.61	1.35 ± 0.22	2.07 ± 0.30	1.44 ± 0.26

Data are represented as mean ± standard deviation.

LME, Lemon Myrtle leaf water extract.

Table S3. Gross necropsy findings of male and female rats treated with LME in the 28-day oral toxicity study.

		Male				Female			
**LME (mg/kg bw/Day)**		**0**	**250**	**500**	**1000**	**0**	**250**	**500**	**1000**
**No. of Rats**		**5**	**5**	**5**	**5**	**5**	**5**	**5**	**5**
**Organ / Finding**									
Spleen									
Nodule	-	5	4	5	5	5	5	5	5
	+	0	1	0	0	0	0	0	0
Lung									
Brown	-	5	5	4	5	5	5	5	5
	+	0	0	1	0	0	0	0	0
Brown patch	-	5	4	5	5	5	5	5	5
	+	0	1	0	0	0	0	0	0
Liver									
Hepatodiaphragmatic nodule	-	5	4	5	5	5	5	4	5
	+	0	1	0	0	0	0	1	0
White patch	-	5	4	5	5	5	5	5	5
	+	0	1	0	0	0	0	0	0
Kidney									
Cyst	-	4	4	5	5	5	5	5	5
	+	1	1	0	0	0	0	0	0
Depression, focal	-	4	3	4	4	5	5	3	5
	+	1	2	1	1	0	0	2	0

LME, Lemon Myrtle leaf water extract.

-: Normal, +: presence of finding

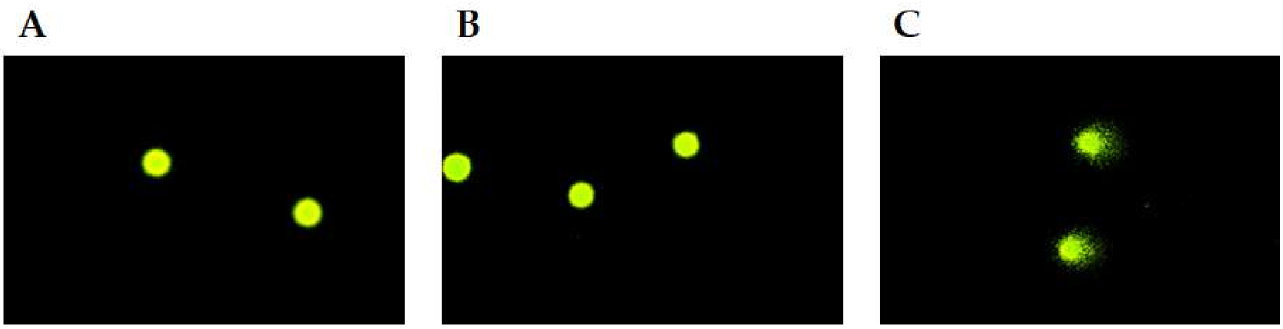

Figure S1. Microscopic images in the liver in the in vivo mammalian alkaline comet assay. (A) Negative control (Distilled Water) groups, (B) LME-2000 mg/kg bw/day groups, and (C) Positive control (Ethyl methanesulfonate) groups.

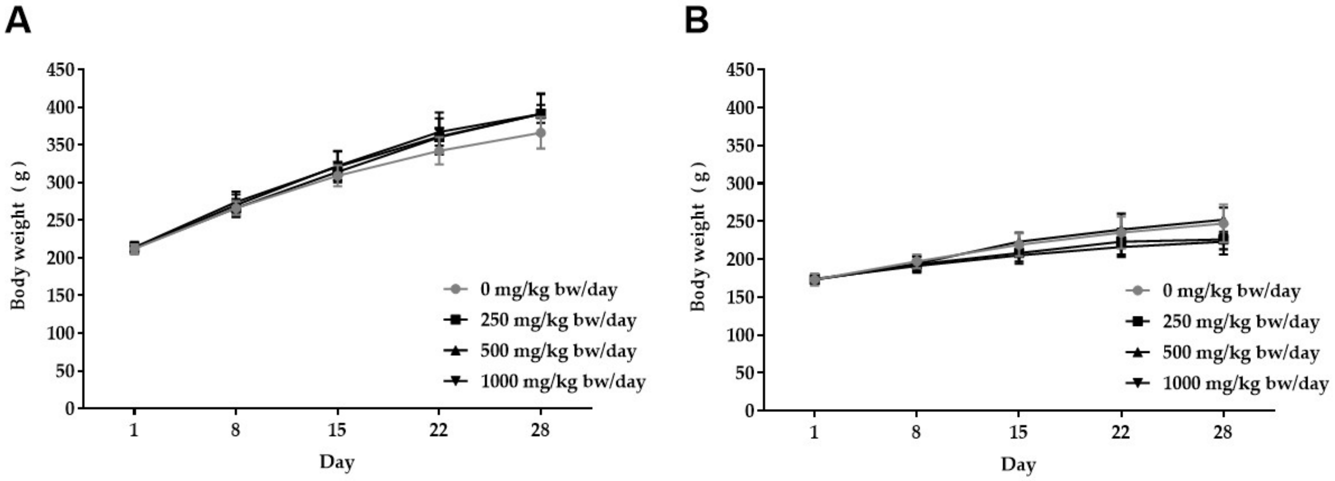

Figure S2. Changes in the body weight of rats. (A) Male and (B) female rats treated with LME in the 28-day oral toxicity study.

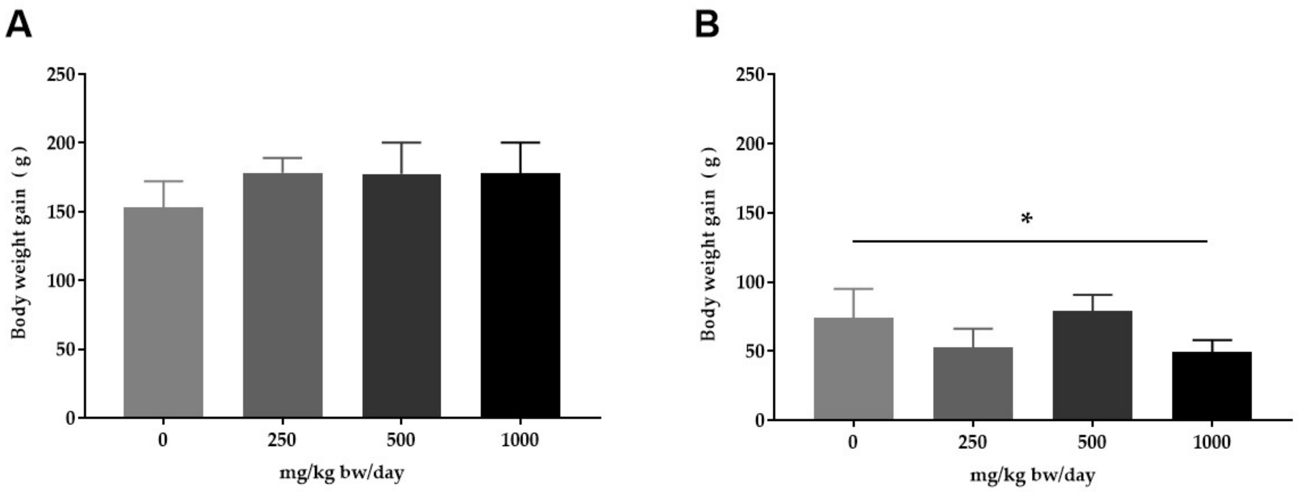

Figure S3. Body weight gains in rats. (A) Male and (B) female rats treated with LME in a 28-day oral toxicity study. Significantly different from the female 0 mg/kg bw/day group (* $p < 0.05$).

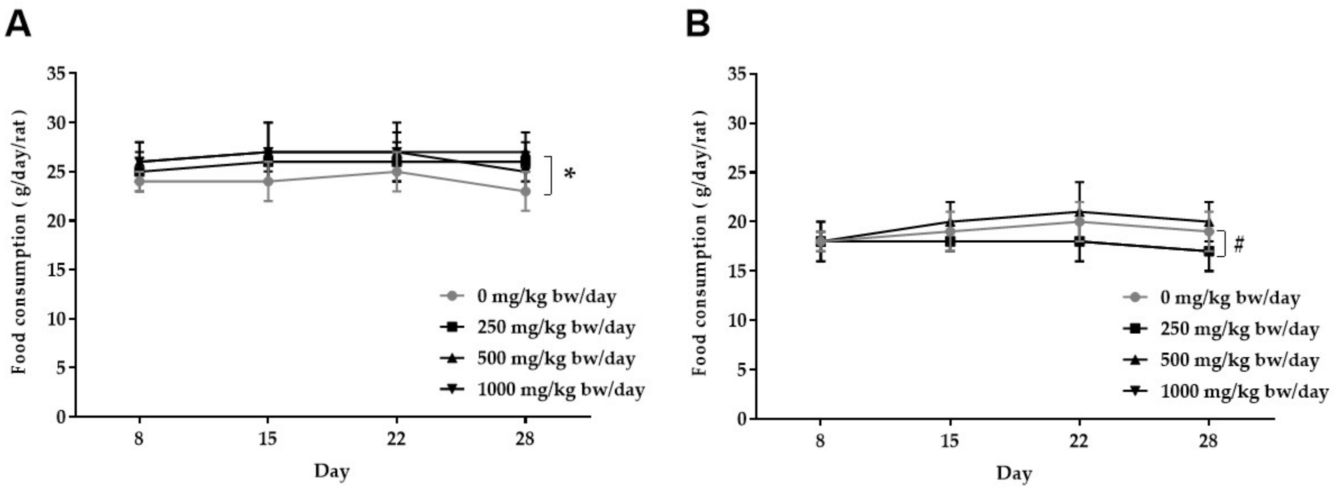

Figure S4. Changes in food consumption among rats. (A) Male and (B) female rats treated with LME in a 28-day oral toxicity study. Significantly different from the 0 mg/kg bw/day group for the same sex (* $p < 0.05$, # $p < 0.05$).
